# Untapped Sources of Dual Resistance to Hessian Fly and Greenbug in Synthetic Hexaploid Wheats

**DOI:** 10.3390/plants12223883

**Published:** 2023-11-17

**Authors:** Subhashree Subramanyam, Harold E. Bockelman, Nagesh Sardesai

**Affiliations:** 1 Crop Production and Pest Control Research Unit, United States Department of Agriculture-Agricultural Research Service (USDA-ARS), West Lafayette, IN 47907, USA; 2 Department of Entomology, Purdue University, West Lafayette, IN 47907, USA; 3 National Small Grains Collection, United States Department of Agriculture-Agricultural Research Service (USDA-ARS), Aberdeen, ID 83210, USA; 4 Corteva Agriscience, Johnston, IA 50131, USA

**Keywords:** *Triticum aestivum*, synthetic hexaploid wheat (SHW), *Mayetiola destructor*, *Schizaphis graminum*, gall midges, aphids, diptera, resistance, susceptibility

## Abstract

The Hessian fly (Hf) and greenbugs (Gb) are major pests of wheat, causing severe economic losses globally. Deploying resistant wheat is the most effective strategy for managing these destructive insects. However, the resistance is not effective against all Hf or Gb biotypes and can impose selection pressure on insects, resulting in the development of virulent biotypes. These challenges must be met through the discovery of new and novel sources of resistance to these pests. Synthetic Hexaploid Wheat (SHW)-developed cultivars are a rich source of resistance against a diverse array of pathogens and pests. In this study, 80 SHW lines were evaluated for their resistance to Hf and Gb under controlled environmental conditions. Of these, a total of 36 SHW lines showed resistance independently to Hf biotype L and Gb biotype E, while 27 lines showed combined resistance to both Hf and Gb. Further, a subset of 10 SHW lines showed resistance to additional Hf biotypes, Great Plains and *vH13*. The identification of SHW lines resistant to multiple insects and biotypes offers an invaluable resource to breeders who are looking to stack resistance traits to develop elite cultivars as a strategy to alleviate economic impacts upon global wheat production.

## 1. Introduction

Wheat (*Triticum aestivum* L.) is the second-most-consumed field grain crop in the world and the third ranked in production in the United States (USDA, 2023, https://www.ers.usda.gov/topics/crops/wheat/ (accessed on 27 September 2023)). However, losses in wheat production due to insect pests are significant, totaling around 40 metric megatons per year [1]. The Hessian fly (Hf; *Mayetiola destructor* [Say]) and the greenbug (Gb; *Schizaphis graminum* [Rondani]), belonging to the orders Diptera (family: Cecidomyiidae) and Hemiptera (family: Aphididae), respectively, are two economically important destructive pests of wheat in the US and around the world, causing losses of hundreds of millions of dollars annually [2,3,4]. Although both these pests vastly differ in their feeding guild, they resemble each other by using an effector-based strategy to establish virulence and modulate host plant physiology. This benefits the developing larvae and leads to host plant susceptibility [5,6,7,8].

The Hessian fly is a gall midge and an obligate parasite of host wheat and triggers a compatible (susceptible plant) or incompatible (resistant plant) interaction. The neonates (newly hatched 1st instar larvae) crawl to the base of the wheat crown from the leaves, where they establish feeding sites. On resistant plants, larvae die within 4–5 days after egg-hatch (DAH) and wheat plants show normal growth [9]. However, on susceptible plants, larvae complete their development within 28–30 days, rendering the plants stunted [10].

Amongst the cereal aphids, greenbug (Gb) is one of the most prevalent pests of wheat. These are small, oval, pale-green aphids with a dark-green stripe running down the middle of the back. Stems and leaves of the plants are fed upon by Gb via piercing-sucking stylets. The stylets are inserted into the phloem sieve elements and the phloem sap is consumed as the food source [11]. Feeding on a wheat plant by Gb causes the production of distinct symptoms that include macroscopic necrotic lesions at feeding sites surrounded by chlorotic zones on leaves within 3–4 days. Gb feeding rapidly induces leaf chlorosis in susceptible wheat plants, leading to the deterioration of plant health and eventual death [12].

The most prevalent integrated pest management strategy—that is economical and environmentally friendly—for controlling Hf and Gb infestation is the use of wheat varieties that harbor resistance genes against different biotypes of the insect pests. A total of 18 biotypes, designated as A to O, GP (Great Plains), *vH9*, and *vH13* have been reported for Hf [13]. While a set of 10 Gb biotypes were found to differentially interact with host plants (designated as A-C, E-K) from 1961 to 1997 [14], 13 new biotypes were collected from wheat, sorghum, rye, and barley accessions from 4 U.S. states [15]. More recently, in 2010, 13 new biotypes were discovered [16], with 6 more in 2016 [17]. To date, 37 Hf resistance genes (designated *H1-H36* and *Hdic*) have been identified in wheat and other wild relatives, with some deployed for commercial production [18,19]. However, Hf are notoriously good at defeating wheat resistance, as deploying single *H* gene cultivars has resulted in the rapid selection of virulent insect biotypes within 6–8 years after cultivar release [20]. For Gb, eight permanently designated resistance genes (*Gb1–Gb8*) and seven genes with temporary designations (*Gba, Gbb, Gbc, Gbd, Gbx1, Gby*, and *Gbz*) have been identified [21,22,23,24,25,26,27]. Of these, only *Gb3* has been widely deployed in breeding programs; however, several Gb biotypes are virulent to this resistance gene [28]. The lack of Hf and Gb resistance genes against the genetic diversity of the biotypes poses a potential threat, especially in the event of an outbreak. Thus, there is an urgent need to identify wheat germplasm with new and novel sources of native resistance that can be integrated into breeding programs and mitigate the devastating effects of these insect pests.

Synthetic Hexaploid Wheat (SHW) are hybrid genotypes (2n = 6× = 42, AABBDD) derived from a cross between durum (*T. turgidum* sp *durum* L.; 2n = 4× = 28, AABB) wheat and wild goat grass (*Aegilops tauschii* Coss.; 2n = 2× =14, DD). SHW shows resistance to a broad spectrum of diseases and pests [29] including Hf and Gb [30,31]. Two genes, *H32* and *Gb7* have been identified from a SHW line (‘W7984′ referred to as Synthetic hereafter), conferring resistance to Hf and Gb, respectively [31,32]. The genes, *Gb3* and *Gb4*, conferring resistance to Gb have also been identified from SHW lines ‘Largo’ (CI 17895) and CItr 17959, respectively [23]. Additionally, SHWs can be directly crossed with the adapted germplasm; hence, it is possible to transfer desirable traits from wild relatives. Therefore, SHW offers a rich resource to mine for additional Hf and Gb insect resistance genes for bolstering native resistance and for use in breeding programs to develop elite cultivars to mitigate the effects of these economically important pests of wheat. In the current study, we evaluated 80 SHW wheat accessions and identified lines showing resistance to Hf and Gb, with some lines showing dual resistance to both the insect pests. These resistant SHW lines offer valuable tools to breeders and farmers for wheat crop management.

## 2. Results and Discussion

**Figure 1:**
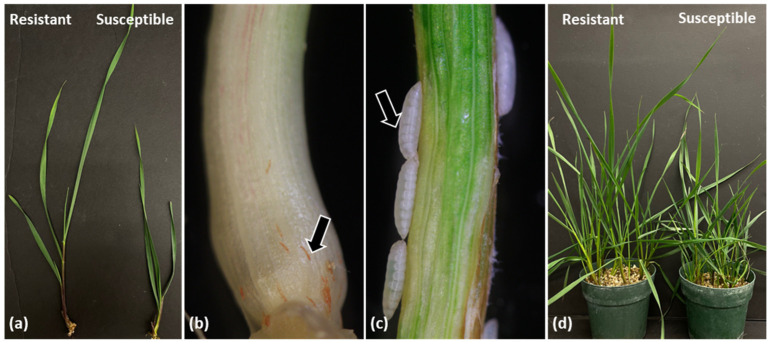
Phenotypic response observed in SHW lines to Hf larval feeding. (**a**) Representative photo of a resistant plant showing normal growth and a susceptible plant showing stunted phenotype; (**b**) Representative resistant plant harboring dead, red 1st instar larvae (arrow); (**c**) Representative susceptible plant harboring white, 2nd instar live, developing larvae (arrow); (**d**) Representative photo of resistant and susceptible SHW lines in pots showing normal and stunted growth, respectively.

The 80 SHW accessions used in this study (Table S1) were screened against the Hf biotype L and assessed visually for either normal or stunted growth resembling the resistant and susceptible control wheat lines, respectively (Figure 1a). All plants showing normal growth were dissected under the microscope to look for dead, red 1st instar larvae (Figure 1b), while randomly selected, stunted plants were dissected to look for live, white 2nd instar larvae (Figure 1c).

The phenotypic response of the 80 SHW lines to Hf biotype L infestation is provided in Table S2. A total of 36 SHW lines showed >70% resistance, with 31 of these showing 100% resistance (Table S2). Wheat lines with >70% resistance to Hf are classified as moderately to strongly resistant [33,34]. The hexaploid wheat differentials included in the study as controls exhibited a susceptible response to Hf biotype L, as expected. Similarly, the positive resistant (Synthetic cultivar harboring the *H32* gene) and negative susceptible (Newton cultivar lacking any *H* gene) controls showed expected responses (Table S2).

Resistance of wheat to Hf involves the gene-for-gene recognition of the insect avirulence gene product by the plant resistance gene product [35]. For example, two Hf biotypes, L and *vH13*, exhibit differences in their interaction with wheat plants harboring the *H13* resistance gene. While the former is avirulent (plant shows resistance phenotype), the latter is virulent (plant shows susceptibility phenotype) when feeding on *H13* harboring plants [36]. Therefore, the virulence or avirulence of a Hf biotype is dependent upon the recognition of the biotype effector by the plant *H* gene. To determine if these SHW lines recognize effectors from multiple Hf biotypes, a subset (17 lines) of SHW lines exhibiting 100% resistance to biotype L were subsequently evaluated against GP and *vH13* Hf biotypes. The two SHW lines, PI 639730 and PI 639732, included within this subset have previously been phenotyped, with the former being resistant to both biotypes and the latter showing resistance to GP but susceptibility to *vH13* [37]. These two SHW lines were therefore used as controls in the screening test against GP and *vH13* Hf biotypes in the current study. The controls showed similar results as documented previously (Table S3) [37]. Of the 15 remaining lines, 12 SHW lines exhibited >70% resistance against both GP and *vH13* flies, while one line (PI 613303) was resistant to GP but susceptible to *vH13* flies (Table S3). All resistant SHW lines from the subset tested with the three biotypes showed normal growth as compared to the susceptible SHW lines that displayed stunting (Figure 1d). Our results indicate that the Hf resistance genes present in the 10 SHW lines (Tables S2 and S3) have the potential to recognize effectors from all three biotypes (L, GP and *vH13*), resulting in these biotypes being avirulent on these wheat lines. Biotype L is a particularly virulent biotype of Hf that represents a significant percentage of field populations from the southeastern and midwestern United States [38]. While GP is considered the least virulent Hf biotype [39], it is a predominant proportion in the majority of field populations from the northwestern US [38]. The *vH13* Hf is virulent on wheat lines that carry the *H13* resistance gene and is one of the best-studied cognate gene combinations between wheat and Hf [40]. Thus, it is likely that the line PI 613303, that was resistant to biotype L and GP but susceptible to *vH13* (Table S3), may carry the *H13* resistance gene. Further genetic characterizations of these SHW lines will be required to determine whether they have new Hf resistance genes or are allelic to existing genes. The importance of identifying resistant lines is highlighted by the results of a study on the relationship between Hf intensity and wheat yields in Oklahoma, in which it was demonstrated that the presence of one larva per tiller over a growing season results in yield loss to the tune of 386 kg/ha [41]. In a separate systematic two-year study on quantitative yield loss in the Pacific Northwest, where GP is predominant, Hf infestation decreased grain value ranging from $133-$176/ha [2]. It is interesting to note that while the well-documented control Synthetic line W7984, harboring the *H32* resistance gene, is resistant to biotype L, it is susceptible to the less virulent GP [31]. In our studies, 15 SHW lines were resistant to GP and 2 were susceptible, indicating a rich diversity in Hf resistance in these lines and making them potential candidates of resistance for use in future breeding programs.

**Figure 2:**
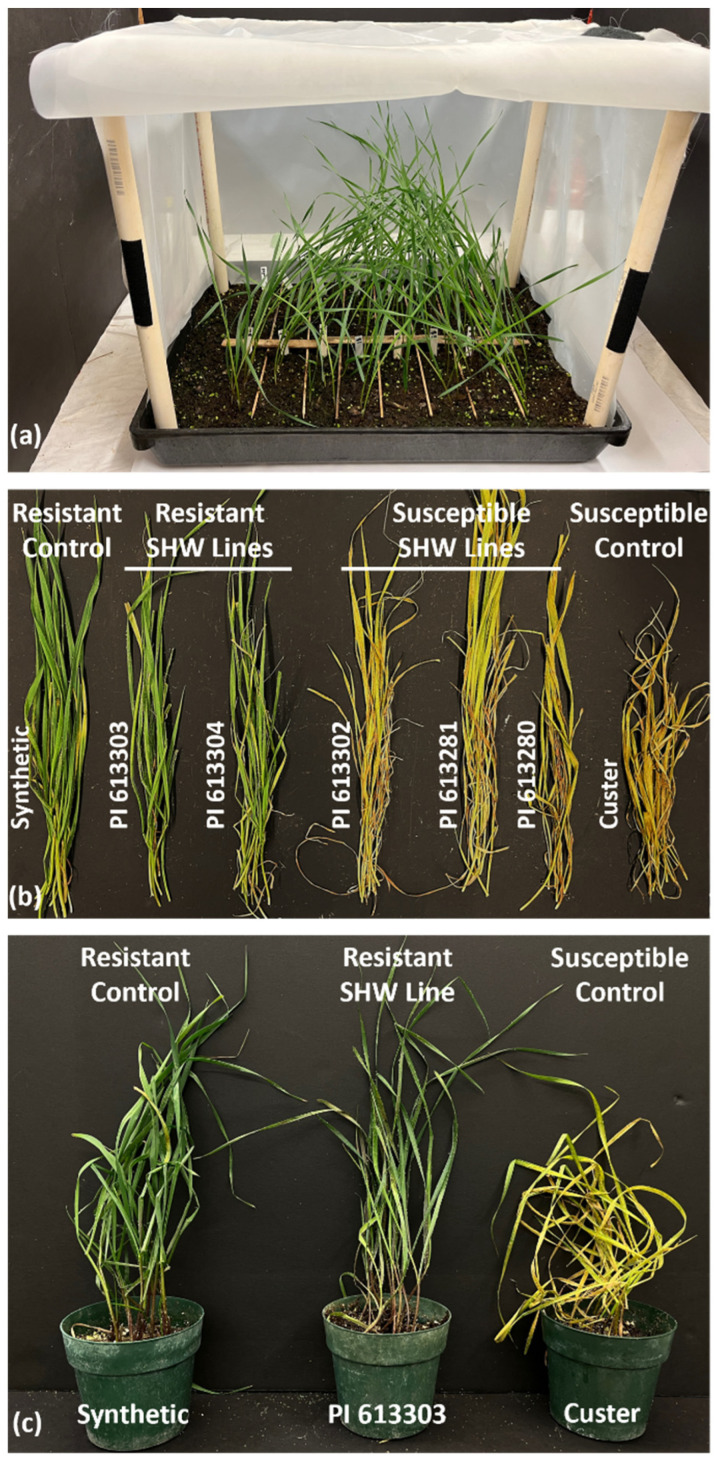
Phenotypic response observed in SHW lines to Gb feeding. (**a**) Representative photo of SHW lines being screened in a flat; (**b**) Representative photo comparing the resistant and susceptible SHW lines with the resistant and susceptible controls in flats; (**c**) Representative photo of pots of a resistant SHW line along with the resistant (Synthetic) and susceptible (Custer) controls.

In addition to Hf, cereal aphids including Gb are important pests of wheat [42]. We evaluated the phenotypic response of the 80 SHW lines for resistance to Gb biotype E infestations in flats (Figure 2a) under controlled environmental conditions. The resistant and susceptible controls behaved as expected, with the former showing 0% chlorosis and the susceptible controls showing 90–100% chlorosis 16 days after infestation (DAI) (Figure 2b). The phenotypic response of the 80 SHW lines to Gb biotype E infestation are shown in Table S4. A total of 36 lines exhibited resistance to Gb biotype E. The susceptible SHW lines in the flats had a distinct chlorotic phenotype that was easily distinguishable from the resistant lines (Figure 2b). To validate the results of the flat test, we subsequently reevaluated the resistance response in a representative subset of 11 Gb biotype E-resistant SHW lines identified from the flat test, in pots. As expected, the pot test validated the flat test results, with all plants for all 11 SHW lines resembling the resistant wheat (Synthetic) control phenotype, unlike the susceptible (‘Custer’) control which showed intense leaf chlorosis (Figure 2c).

The leaf chlorosis observed in aphid-infested, susceptible wheat is due to a loss of photosynthetic pigments [43,44]. Hence, the assessment of the total chlorophyll (Chl) levels and the quantification of photosynthetic pigments (Chl*a*, Chl*b* and carotenoids) in the leaf tissue of a stressed plant is an important indicator of senescence and plant health [45,46]. A subset of five SHW Gb biotype E-resistant lines was used to measure the total Chl levels from Leaf 1 and Leaf 2 between uninfested and infested plants 11 DAI, using a SPAD meter.

**Figure 3:**
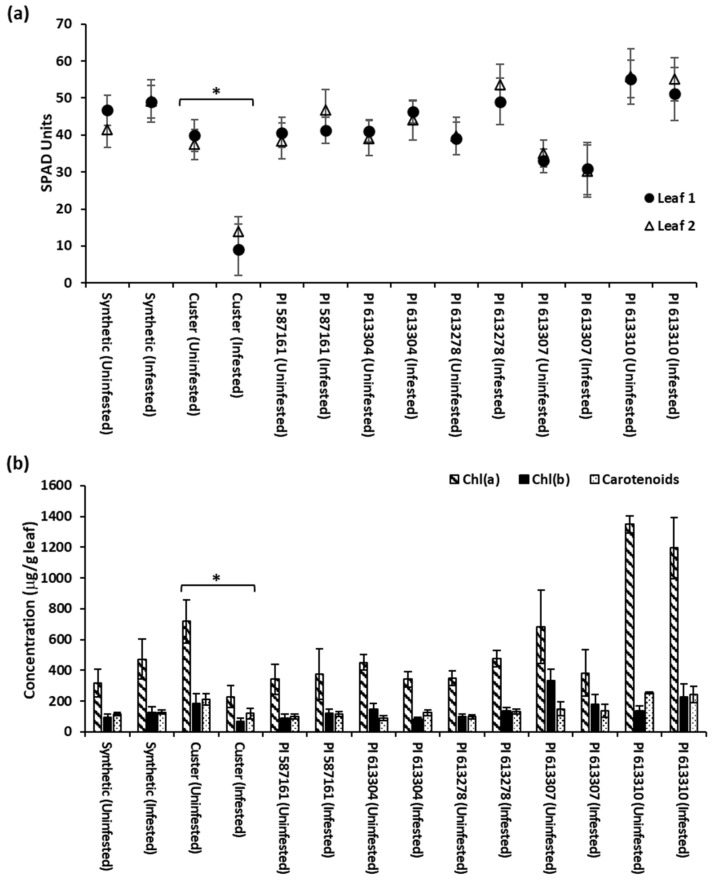
Chlorophyll content in representative wheat lines infested or uninfested with Gb biotype E. (**a**) Total chlorophyll content was measured for Leaf 1 and Leaf 2 of Gb-infested and uninfested representative wheat lines. Values are represented as SPAD units. Pairs of Gb-uninfested and infested wheat lines with statistically significant differences are denoted by “*”. The *p* values for these pairs are <0.0001 for both Leaf 1 and Leaf 2. (**b**) Individual Chlorophyll a [Chl(a)], Chlorophyll b [Chl(b)] and carotenoid concentrations were estimated in leaves of Gb-infested and uninfested wheat lines. Levels are represented as µg/g leaf fresh weight. Pairs of Gb-uninfested and infested wheat lines with statistically significant differences are denoted by “*”. The *p* values for these pairs are <0.0001, 0.0045, and <0.0001 for Chl(a), Chl(b), and Carotenoids, respectively.

Figure 3a shows the total Chl levels as SPAD units in Leaf 1 and Leaf 2 for each Gb-infested and uninfested SHW line, along with the resistant and susceptible controls. None of the resistant SHW lines showed decreased Chl levels in either of the Gb-infested leaf samples as compared to their uninfested plants (Figure 3a). The only cultivar that showed significantly (*p* < 0.0001) lower levels of total Chl was the Gb-infested susceptible control wheat line, Custer (Figure 3a). The Gb-infested Custer showed mean total Chl levels as low as 9 and 13.9 SPAD units in Leaf 1 and Leaf 2, respectively, as compared to the uninfested control plants that showed total Chl levels of 39.8 and 37.4 SPAD units (Figure 3a). All other uninfested and Gb-infested resistant Synthetic and SHW lines showed total Chl levels ranging from 30–55 SPAD units. Our results clearly indicate that the resistant SHW lines are able to tolerate heavy aphid infestation and probing without any significant loss in Chl, unlike the susceptible control, in which aphid-infestation negatively correlates with the total Chl levels as clearly evidenced by the yellowing of the leaves (Figure 2b,c). Similar results for the significant reduction of total Chl levels in susceptible wheat compared to other Synthetic wheat varieties following Gb-infestation have been seen [30,47].

Severe chlorosis in plants due to aphid feeding also affects plant photosynthetic efficiency [48,49,50]. The primary pigments in plants that harvest light energy for photosynthesis are Chl*a* and Chl*b*, as well as carotenoids that absorb light between 400 and 500 nm and transfer energy to chlorophyll molecules [51]. Our results showed no significant difference in Chl*a* (*p* > 0.05) and Chl*b* (*p* > 0.05) levels in Gb-infested SHW lines as compared to the levels observed in their uninfested controls (Figure 3b). However, significantly lower levels for Chl*a* (*p* < 0.0001) and Chl*b* (*p* = 0.0045) were observed in the susceptible control wheat line, Custer, infested with Gb as compared to the uninfested control (Figure 3b). Similarly, we also observed significantly lower levels of carotenoids (*p* < 0.0001) in Gb-infested susceptible wheat Custer compared to uninfested plants, whereas aphid-infested resistant SHW lines and the resistant control Synthetic had levels similar (*p* > 0.05) to their uninfested controls (Figure 3b). Carotenoids are important in photosynthesis and act as accessory light harvesters and harmful quanta quenchers in higher plants [52]. A reduction in carotenoids was found to be detrimental to wheat photosynthesis due to feeding by the Russian wheat aphid, *Diuraphis noxia* [50]. These results demonstrate that the SHW lines categorized as resistant are able to maintain their photosynthetic efficiency and tolerate aphid probing. So far, out of the 8 permanently designated Gb resistance genes, three (*Gb3, Gb4* and *Gb7*) have been identified from synthetic hexaploid wheat lines Largo, CI 17959, and W7984, respectively [23,32,53,54,55]. Therefore, it is clear that SHW lines are a rich source of Gb resistance genes that can be deployed in breeding programs by identifying, mapping the resistance gene(s), and further developing tightly linked flanking markers for marker-assisted selection. Further studies are required to assess whether the Gb resistance genes in the 36 SHW lines identified in the current study are allelic to any of the known Gb resistance genes or are novel resistance genes.

Combined resistance within a wheat line to multiple insect pests is a useful trait for breeders who are looking to develop wheat lines with molecular stacks and pyramid resistance genes that are effective against diverse pests and biotypes. Our phenotypic evaluation showed a total of 27 SHW lines having dual resistance to both the Hf biotype L and the Gb biotype E (Table 1). Additionally, a subset of 12 SHW lines evaluated also showed resistance to two different Hf biotypes, GP (11) and *vH13* (10). These lines with dual resistance hold great promise for preventing economic losses that can exceed $100 million annually in the Great Plains region of the US due to greenbug feeding alone [3]. Similar combined resistance to multiple pests has been documented previously. Three other SHW lines that are resistant to the Syrian Sunn pest (*Eurygaster integriceps* Puton) are also highly resistant (85–100%) to Hf from Morocco [34]. Resistance to multiple pathogens and insects is reported in several different SHW lines [56,57]. In addition to Hf and Gb resistance, 65 of the SHW lines tested here are reported to show additional resistance to other biotic stresses including karnal bunt, spot blotch, tan spot, stem rust, *Fusarium* head blight, and the Russian wheat aphid (Table S1). Eighteen SHW lines have combined resistance to Hf, Gb, and Russian wheat aphids, while 8 lines show combined resistance to Hf, Gb, and karnal bunt. Thus, these SHW lines are new, untapped resources that have a great potential for future insect- and pathogen-resistance gene stacking breeding programs.

## 3. Materials and Methods

### 3.1. Insect and Plant Material

The Hessian fly stocks, biotype L, *vH13*, and GP, used in the current study were maintained in cold (4 °C) storage in a diapause stage as described by Sosa and Gallun [58]. The greenbug biotype E stock was maintained on wheat (*Triticum aestivum*) cultivar ‘Newton’ in a growth chamber (Revco, Thermo Fisher Scientific, Waltham, MA, USA) at 22 °C with 16:8 h light/dark cycle. Both the insect pests were maintained in the USDA-ARS Crop Production and Pest Control Research Unit in West Lafayette, IN, USA.

A total of 80 *Aegilotriticum* spp. SHW lines used in this study (Table S1) were obtained from the National Small Grains Collections (NSGC), Aberdeen, ID. Hexaploid wheat (*Triticum aestivum*) lines ‘Monon’ [59], ‘Magnum’ [60], ‘Caldwell’ [61], and ‘Seneca’ [62], harboring the Hessian fly resistance genes *H3, H5, H6* and *H7H8* [63,64], respectively, were used as differentials to confirm the purity of Hf biotype L and GP stocks. All four differential wheat lines are susceptible to biotype L and resistant to GP. To confirm the purity of Hf *vH13* stocks, hexaploid wheat lines ‘Molly’ (harboring the *H13* resistance gene) and ‘Iris’ (harboring the *H9* resistance gene) served as susceptible and resistant controls, respectively. The hexaploid wheat accession ‘W7984′ (harboring the *H32* and *Gb7* Hf and Gb resistance genes, respectively, and referred to throughout as Synthetic) was used as the resistant control for both the insect pests. The wheat lines Newton and Custer were used as susceptible controls for Hf and Gb insects, respectively.

### 3.2. Evaluation of Hf Resistance in SHW Lines

#### 3.2.1. Screening with Hf Biotype L

For each of the SHW lines, 15 seeds were planted in flats [36]. The plants were grown in a controlled growth chamber (Percival Scientific, Perry, IA, USA) set at 20 °C with 14:10 h light/dark cycle. At the 2-leaf stage, the flats were covered with cheesecloth and the plants were infested with Hf biotype L, as described previously [36]. To confirm the purity of the fly material in each flat, 4-inch pots containing four differential hexaploid wheat lines were grown as described above and placed under the cheesecloth along with the flats. The wheat lines Synthetic and Newton were also planted in 4-inch pots and placed under the cheesecloth along with the flats as resistant and susceptible plant controls, respectively. Eight days after egg hatch (DAH), the phenotypic response of each plant was recorded. The plants showing normal or stunted growth were recorded as resistant or susceptible plants, respectively. All plants from the SHW lines showing normal growth were dissected to confirm the presence of dead, red larvae (1st instar). The number of 1st instar (dead, red) larvae was counted in all the resistant plants (showing normal growth) for each SHW line. If no larvae were found, the plants were categorized as escapes and excluded from the data analysis. Randomly selected susceptible plants showing stunted growth from each SHW line were also dissected to confirm the presence of 2nd or 3rd instar (white, live and developing) larvae. If >70% of plants of a line had only dead, red larvae, the SHW accession was categorized as being resistant to Hf biotype L.

#### 3.2.2. Screening with Hf *vH13* and GP

A representative subset of 17 SHW lines that showed > 70% resistance to Hf biotype L were used for further screening with the Hf biotypes *vH13* and GP, as described above. For screening with *vH13*, wheat lines Iris and Molly were included as resistant and susceptible controls, respectively [65]. To confirm the purity of GP, 4-inch pots containing the four differential hexaploid wheat lines were grown as described above and infested along with the SHW lines.

### 3.3. Evaluation for Gb Resistance in SHW Lines

#### 3.3.1. Screening with Gb Biotype E

Flats (15” × 21”) containing Pro-Line C/20 Growing Mix (Jolly Gardner Products Inc., Poland Spring, ME, USA) were prepared and 10 rows were pressed into the soil. Seeds (10 per wheat line) for 8 lines each were planted in the top and bottom half of the flat. The plants were grown in a growth chamber (Revco) set at 22 °C with 16:8h light/dark cycle. When the plants reached the 2-leaf stage, they were infested with Gb biotype E (at least 25–30 aphids per plant) and covered with a nylon mesh cage. The wheat lines Synthetic and Custer were included as resistant and susceptible controls, respectively. Sixteen days after infestation (DAI), the plants were evaluated using the visual appearance of leaf chlorosis in comparison to the resistant and susceptible controls included in each flat test.

To confirm the results obtained from the flat test, a representative subset of 11 resistant SHW lines was retested in pots. Five seeds per resistant SHW line were planted in 4-inch pots and, when the plants reached the 2-leaf stage, they were infested with Gb biotype E as described above. The phenotypic response of the SHW lines, along with resistant and susceptible control lines, was recorded on 16 DAI.

#### 3.3.2. Total Chlorophyll Estimation

The Soil Plant Analysis Development (SPAD) meter (SPAD-502, Konica-Minolta, Chiyoda City, Japan) was used to measure the leaf’s total chlorophyll levels in a representative subset of five uninfested and Gb-infested SHW resistant lines (PI 587161, PI 613304, PI 613278, PI 613307 and PI 613310), along with the uninfested and infested resistant (Synthetic) and susceptible (Custer) control lines, on 11 DAI. Three plants of each wheat line for Gb-uninfested and infested treatments were used for the measurements. Five independent SPAD readings were taken per leaf for Leaf 1 and Leaf 2 from each plant. Statistical analysis of the difference in the means of SPAD units between infested and uninfested leaves for both leaves of each wheat line sampled was carried out using the one-sided Tukey pairwise comparison (JMP Pro Ver. 16, SAS Institute Inc., Cary, NC, USA).

#### 3.3.3. Estimation of Photosynthetic Pigments Chlorophyll a, Chlorophyll b, and Carotenoids

The concentration of photosynthetic pigments Chlorophyll a (Chl*a*), Chlorophyll b (Chl*b*), and Carotenoids was measured in the same Gb-infested and uninfested subset of five Gb-resistant SHW lines chosen for SPAD analysis, along with the resistant (Synthetic) and susceptible (Custer) control as described in Wang et al. [50]. Briefly, 0.3 g of leaf tissue was ground in liquid nitrogen with a mortar and pestle and 3 mL of 80% acetone was added. The suspension was centrifuged at 6000× *g* for 10 min to remove insoluble plant tissue. The supernatant was collected and diluted with 80% acetone to adjust the absorbance readings at 663 nm between 0.1 and 1.5. The absorbance was measured at 470, 646, and 663 nm using the Ultrospec 3300 *pro* UV/Visible spectrophotometer (Biochrom Ltd., Cambridge, UK). The concentration of Chl*a*, Chl*b*, and carotenoids was calculated as microgram per gram of fresh wheat leaf tissue, following the equation described by Bertrand and Shoefs [66]. The statistical analysis of the photosynthesis pigment concentrations between the infested and uninfested leaves of each wheat line tested was carried out using the one-sided Tukey pairwise comparison (JMP Pro Ver. 16).

## Figures and Tables

**Table 1:** SHW lines showing resistance (R) or susceptibility (S) to multiple Hessian fly (Hf) biotypes and Greenbug (Gb) biotype E.

S. No	Accession	Hf Biotype L	Hf Biotype GP	Hf Biotype *vH13*	Gb Biotype E
Origin: Mexico, Mexico City					
1	PI 587161	R	Nd	Nd	R
2	PI 587162	R	Nd	Nd	R
3	PI 587163	R	R	R	R
4	PI 613303	R	R	S	R
5	PI 613304	R	Nd	Nd	R
6	PI 613305	R	Nd	Nd	R
7	PI 613309	R	R	R	R
8	PI 613311	R	Nd	Nd	R
9	PI 648478	R	R	R	R
10	PI 648480	R	S	S	R
11	PI 648481	R	Nd	Nd	R
12	PI 648482	R	R	R	R
13	PI 648483	R	Nd	Nd	R
14	PI 648484	R	R	R	R
15	PI 648485	R	Nd	Nd	R
16	PI 648486	R	R	R	R
17	PI 648497	R	R	R	R
18	PI 648501	R	R	R	R
19	PI 648509	R	Nd	Nd	R
20	PI 648510	R	R	R	R
21	PI 648511	R	Nd	Nd	R
22	PI 648513	R	Nd	Nd	R
23	PI 648517	R	Nd	Nd	R
24	PI 648518	R	Nd	Nd	R
25	PI 648520	R	Nd	Nd	R
26	PI 648522	R	Nd	Nd	R
Origin: United States, North Dakota					
27	PI 639730	R	R	R	R

Nd: Not determined.

## Data Availability

The data presented in this study are available in enclosed Figures, Tables and Supplementary Materials.

## Supplementary Materials

The following supporting information can be downloaded at: https://www.mdpi.com/article/10.3390/plants12223883/s1, Table S1: Synthetic Hexaploid Wheat lines evaluated for resistance against Hessian fly (biotype L) and Greenbug (biotype E); Table S2: Phenotypic response of Synthetic Hexaploid Wheat lines to Hessian fly biotype L; Table S3: Phenotypic response of Synthetic Hexaploid Wheat lines to Hessian fly biotypes GP and *vH13*; Table S4: Phenotypic response of Synthetic Hexaploid Wheat lines to Greenbug (Gb) biotype E.
